# *TOX* and *CDKN2A/B* Gene Polymorphisms Are Associated with Type 2 Diabetes in Han Chinese

**DOI:** 10.1038/srep11900

**Published:** 2015-07-03

**Authors:** Fengjiang Wei, Chunyou Cai, Shuzhi Feng, Jia Lv, Shen Li, Baocheng Chang, Hong Zhang, Wentao Shi, Hongling Han, Chao Ling, Ping Yu, Yongjun Chen, Ning Sun, Jianli Tian, Hongxiao Jiao, Fuhua Yang, Mingshan Li, Yuhua Wang, Lei Zou, Long Su, Jingbo Li, Ran Li, Huina Qiu, Jingmin Shi, Shiying Liu, Mingqin Chang, Jingna Lin, Liming Chen, Wei-Dong Li

**Affiliations:** 1 Research Center of Basic Medical Sciences, Tianjin Medical University, Tianjin, 300070, China; 2 Tianjin General Hospital, Tianjin Medical University, Tianjin, 300052, China; 3 Metabolic Diseases Hospital, Tianjin Medical University, Tianjin, 300070, China; 4 Eye Hospital, Tianjin Medical University, Tianjin, 300384, China; 5 Tianjin People’s Hospital, Department of Endocrinology, Tianjin, 300191, China

## Abstract

To study associations between type 2 diabetes (T2DM) candidate genes and microvascular complications of diabetes (MVCDs), we performed case-control association studies for both T2DM and MVCDs in Han Chinese subjects. We recruited 1,939 unrelated Han Chinese T2DM patients and 918 individuals with normal blood glucose levels as nondiabetic controls. Among T2DM patients, 1116 have MVCDs, 266 have a history of T2DM of >10 years but never developed MVCDs. Eighty-two single-nucleotide polymorphisms (SNPs) in 54 candidate genes were genotyped. Discrete association studies were performed by the PLINK program for T2DM and MVCDs. Significant associations were found among candidate gene SNPs and T2DM, including rs1526167 of the *TOX* gene (allele A, *P *= 2.85 × 10^−9^, OR = 1.44). The SNP rs10811661 of the *CDKN2A/B* gene was also associated with T2DM (allele T, *P *= 4.09 × 10^−7^, OR = 1.36). When we used control patients with >10 years of T2DM history without MVCD, we found that the G allele of SNP rs1526167 of the *TOX* gene was associated with MVCD (nominal *P *= 4.33 × 10^−4^). In our study, significant associations were found between *TOX* and *CDKN2A/B* gene SNPs and T2DM. The *TOX* polymorphism might account for the higher risk of T2DM and the lower risk of MVCDs in the Han Chinese population.

The prevalence of type 2 diabetes (T2DM) has increased dramatically in China in recent years^1^,^2^. Many T2DM genes found in European populations have been replicated in Han Chinese; however, many top candidate genes (including *TCF7L2*) have shown only moderate associations with T2DM in East Asian populations. On the other hand, many Asian-specific T2DM genes have been identified in recent studies^3^.

With the remarkable rise worldwide in the prevalence of diabetes, an increase in patients suffering from microvascular complications of diabetes (MVCDs) will be inevitable. Diabetic nephropathy (DN) and diabetic retinopathy (DR), two common MVCDs, are leading causes of end-stage renal failure and blindness in diabetes patients^4^,^5^. Epidemiology data for MVCDs are relatively limited in China, although a recent study showed a much lower prevalence of DR in the Beijing area than in European populations^6^.

Although previous studies have shown that the duration of diabetes and plasma glucose levels are MVCD risk factors^7^,^8^, some diabetes patients with severe hyperglycemia never develop MVCDs. In contrast, many diabetes patients with well-controlled blood glucose suffer from MVCDs^9^. Many studies have found family aggregation of MVCDs and significantly increased risk in siblings, suggested that genetic factors play an important role in the etiology of MVCDs^10^,^11^,^12^,^13^,^14^. Although many association studies have identified genes related to MVCDs^15^, the search for MVCD susceptibility genes was less successful than for T2DM susceptibility genes, partially because of un-diagnosed MVCDs and lack of appropriate normal controls.

It is possible that Han Chinese–specific gene variants are associated with T2DM and/or MVCDs. In this study, we aimed to find T2DM and MVCD candidate genes in Han Chinese T2DM patients by studying associations between single-nucleotide polymorphisms (SNPs) of candidate genes and T2DM/MVCDs.

## Results

Using 1,939 T2DM patients as cases and 918 nondiabetic individuals (>57 years old, with normal blood glucose) as controls, analyses for T2DM showed association between candidate gene SNPs and T2DM (Table 1), including rs10811661 of the *CDKN2A/B* gene (allele T, *P* = 4.09 × 10^−7^, OR = 1.364, 95% CI = 1.209–1.538), rs1526167 of the *TOX* gene (allele A, *P* = 2.85 × 10^−9^, OR = 1.440, 95% CI = 1.276–1.624), rs4402960 of the *IGF2BP2* gene (allele T, *P* = 7.56 × 10^−4^, OR = 1.352, 95% CI = 1.134–1.612), rs6856526 of the *LPHN3* gene (allele C, *P* = 1.33 × 10^−3^, OR = 1.522, 95% CI = 1.176–1.970), rs13266634 of the *SLC30A8* gene (allele C, *P* = 4.56 × 10^−3^, OR = 1.282, 95% CI = 1.080–1.532), and rs7756992 of the *CDKAL1* gene (allele G, *P* = 8.21 × 10^−4^, OR = 1.246, 95% CI = 1.095–1.418). The *TOX* gene SNP rs1526167 association reached genome-wide association level (*P *< 5 × 10^−8^), while the *CDKN2A/B* SNP rs10811661 was significant after Bonferroni correlation (*P *< 1.22 × 10^−4^ for 82 SNPs and 5 binary traits, 6.1 × 10^−4^ for 82 SNPs and 1 binary trait).

In MVCD association studies, DN, DR, proliferative retinopathy (PDR), and MVCD patients were selected as cases, and patients with >10 years T2DM history, without DR or DN, were chosen as controls. Association analyses of dichotomous variables showed that rs1526167 of the *TOX* gene was associated with DN (*P* = 0.0011, OR = 1.470, 95% CI = 1.166–1.854), DR (*P* = 0.0082, OR = 1.412, 95% CI = 1.093–1.825), and MVCD (*P* = 4.33 × 10^−4^, OR = 1.498, 95% CI = 1.195–1.878). However, the at-risk allele for MVCDs was G, rather than A for T2DM. The T allele of rs10811661 of the *CDKN2A/B* gene was associated with DR (*P* = 0.037, OR = 1.314, 95% CI = 1.016–1.698), PDR (*P* = 0.026, OR = 1.708, 95% CI = 1.063–2.743), and MVCD (*P* = 0.025, OR = 1.292, 95% CI = 1.032–1.617). The G allele of rs4402960 of the *IGF2BP2* gene was associated with DN (*P* = 0.0092, OR = 1.499, 95% CI = 1.104–2.034) and MVCD (*P* = 0.0094, OR = 1.481, 95% CI = 1.100–1.995). The T allele of rs12102171 of the *SMAD3* gene was associated with DR and MVCD (*P* = 0.027, and 0.045, respectively). The A allele of the *ESR1* gene was associated with DR and PDR (*P* = 0.041 and 0.033, respectively) (Table 2). None of these nominal associations for MVCDs remained significant after Bonferroni corrections.

Four SNPs of the *TOX* gene were genotyped (Table 3), rs1526167 was not in linkage disequilibrium with other 3 SNPs (Supplement Table 2, Supplement Figures 1 and 2). The SNP rs17304270 of the TOX gene gave marginal association with diabetic nephropathy: allele “A”, OR = 1.514 (1.003–2.285), *P* = 0.047.

## Discussion

To date, more than 30 genes have been identified as reaching the genome-wide significance threshold (*P *< 5 × 10^−8^) for T2DM; 10 of these were replicated in the Han Chinese population^16^. More than 60% of T2DM genes found in East Asian genome-wide association studies (GWAS) were replicated in Han Chinese^17^, compared with approximately 30% of European population T2DM genes. Compared with Caucasians, the Han Chinese and East Asian populations are more insulin resistant, are more centrally obese, and develop T2DM more readily even with moderate increases in body mass index^18^,^19^,^20^. Thus, seeking T2DM genes in Han Chinese would help us to better understanding the global genetic background of T2DM.

In this study, as candidate genes we chose 1) genes related to T2DM, obesity, or insulin resistance found by previous GWAS and 2) genes related to glucose and lipid metabolism, insulin secretion, or MVCD, such as the HIF1α/-VEGF pathway.

A significant association (*P* = 2.85 × 10^−9^) between a *TOX* gene SNP and T2DM was identified for the first time by this study. In addition, four previous reported T2DM genes, *CDKN2A/B*, *IGF2BP2*, *SLC30A8*, and *CDKAL1*, showed moderate to strong associations with T2DM. We did not replicate associations with several well-known T2DM genes, including *TCF7L2*, *FTO*, *IRS1*, and *KCNQ1*, in our Han Chinese population (In our data set, the *FTO* gene SNPs yielded some associations with insulin resistance related phenotypes in quantitative analyses. Data not shown). Potential reasons for this failed replication may include genetic heterogeneity, low allele frequencies of tested SNPs in Han Chinese (e.g., the MAF of *TCF7L2* gene SNP rs7903146 was 0.046), less covered genes (i.e., too few SNPs were genotyped for certain candidate genes), and the relatively small sample size. We performed a gene-specific power calculations for genes *IRS1, TCF7L2, KCNQ1,* and *FTO* using real minor allele frequencies (MAF) in our data set (Supplement Table 1). Except *TCF7L2*, we have adequate power (>80%) to detect associations at the type I error rate (α) = 0.05 when genotype relative risk (GRR) >1.4. We have limited power when GRR ≤ 1.2, it could be the reason of some failed replications for T2DM candidate genes in our study.

Compared with studies of T2DM, GWAS and association studies of MVCD are relatively limited^21^,^22^,^23^,^24^,^25^,^26^. So far about half a dozen of GWAS were performed for MVCD (including 3 in East Asian populations), although none of the MVCD associations reached the genome-wide significance threshold of *P *< 5 × 10^−8^. In order to subtract the influence of T2DM, we selected as MVCD controls subjects with >10 years history of T2DM who never developed DN or DR. We had only 106 PDR patients in the study, but we still achieved moderate nominal *P* values for *CDKN2A/B* and *ESR1* gene SNPs. Recently, Sheu *et al.*^22^ performed a GWAS for DR in a Chinese population, several loci was associated with DR, although no genome wide association with *P *< 1 × 10^−7^ was reached. We have not gotten a chance to test their findings in our population, on the other hand, the *TOX* and *CDKN2A/B* polymorphisms were not among their top associations. We also tested candidate genes for DN that identified by GWAS in African Americans^21^,^27^, including *MYH9, SFI1,* and *LIMK2*, but no association was found for MVCD. Given the sample size of our non-MVCD T2DM controls (266), we only have moderate power for DN and DR association studies. For PDR, the detection power was very limited. On the other hand, we have *MYH9* and *SFI1* gene well covered in our study (8 SNPs for MYH9 and 3 for SFI1, D’ > 0.6), we have >80% power to detect association for MVCD, DN, and DR when GRRs were more than 1.40, 1.42, 1.47, respectively (alpha = 0.05, dominant model). Compare with T2DM, our power for detection of MVCD associations was moderate, mainly because of the limited sample size of the control group. We cannot rule out the *MYH9* and *SFI1* associations that found in African Americans, although population heterogeneity could be a major reason of the difference between Han Chinese and African Americans.

In this study, SNPs in *TOX* and *CDKN2A/B* genes yielded the most significant associations for both T2DM and MVCD. Interestingly, the A allele of the *TOX* gene SNP rs1526167 was the “at-risk” allele in T2DM and the “protection” allele for MVCD. Recent research carried out in Beijing (very close to Tianjin, where our subjects were collected) showed that the prevalence of NPDR was 18.6% in Han Chinese T2DM patients^6^, compared with 28.5% in U.S. T2DM patients, based on NHANES 2005–2008 data^28^. Prevalence of DR was quite different among U.S. ethnic groups and was higher in African Americans than in European Americans^28^. To determine whether the “protection” allele of the *TOX* gene accounts for the lower DR prevalence in Han Chinese MVCD patients, studies with larger sample sizes are needed.

The *CDKN2A/B* gene is located in the chromosome 9p21 region, which has been highlighted as the strongest genetic susceptibility locus for cardiovascular disease (CVD)^29^,^30^ and linked to other conditions such as T2DM^30^,^31^, Alzheimer’s disease^32^, glaucoma^33^, and periodontitis^34^. Interestingly, the region of the chromosome associated with CVD and diabetes was previously considered a gene desert. Previous studies found that rs10811661 has a strong correlation with T2DM in French^35^, Japanese^36^, and Chinese^37^ populations. The SNP rs10811661 locates ~100 kb upstream of *CDKN2A/B*, which has been shown to associate with downregulation of antisense noncoding RNA in *INK4* locus (*ANRIL*) expression^38^. The *ANRIL* methylates histone H3K27 by interacting with polycomb proteins, therefore suppresses the expression of *INK4a* (i.e. *CDKN2A*)^39^. Recently, a large sample sized candidate gene association study for T2DM was carried out in Chinese population^40^, 8 T2DM related genes from previous GWASs were replicated. The *CDKN2A/B* region SNP, rs10811661, yielded the most significant association (P = 1.11 × 10^−8^). The SNP was also associated with many quantitative glycemic traits.

At present, more studies have been done on the correlation between *CDKN2A/B* and the pathogenesis of T2DM than on the relationship between *CDKN2A/B* and MVCD^41^,^42^,^43^. In the present study, we found associations with both T2DM and MVCD for rs10811661, and the allele T contributed to the increased risk for both diseases. Although associations on the *CDKN2A/B* (*ANRIL*) locus were well documented, the mechanism by which this locus affects susceptibility for T2DM and MVCD remains to be investigated.

The *TOX* gene association for T2DM was first discovered in the present study. The *TOX* gene, a protein-coding gene located in human chromosome 8, is one of the *TOX* high-mobility-group proteins. In the present study, we tested 4 SNPs of the *TOX* gene, rs1526167, rs2726557, rs11777927, and rs17304270. The SNP rs1526167 was not in linkage disequilibrium with other 3 SNPs (Supplement Table 2), it could be the reason for the lack of association of T2DM with those 3 SNPs. The SNP rs17304270, however, was nominally associated with diabetic nephropathy.

The history of the SNP rs1526167 was a little complicated. The SNP rs1526167 located 15Kb downstream of the *TOX* gene and 1Kb upstream of an unknown function mRNA *DL491802*. The SNP was first identified by Perlegen Sciences. At the time of the International HapMap Project, that SNP was not included. It could be the reason why rs1526167 was not included in most main stream genome-wide SNP genotyping panels. We checked the LD pattern (in r^2^) of the chr 8: 59,830,000–59,920,000 region in the HapMap for both Chinese (CHB) and Caucasian (CEU) populations (Supplement Figures 1 and 2): the SNP rs1526167 was located between rs10090702 and rs2726588 (indicated by arrows), and it was located in a separate haplotype block, not in LD with SNPs in the *TOX* gene coding region and introns.

Although there was no rs1526167 association reported for T2DM, there were some associations found on that SNP for obesity and metabolic syndrome related phenotypes. In a published US patent application (Pub No: US 2006/0177847 A1, Pub Date: Aug 10, 2006), Cox *et al.* found the *TOX* gene polymorphism and other 27 DNA sequence variations were associated with Olanzapine treatment emergent weight gain and “metabolic syndrome” in a 1.7 million SNPs genome association study. After checking the DNA sequence provided by the patent application, rs1526167 was among the candidate gene SNPs to screen Olanzapine treatment emergent weight gain and other related traits.

The SNP rs1526167 was associated with obesity in European American extremely obese trios by a transmission disequilibrium test (TDT): *P* = 2.2 × 10^−5^; all probands had BMI >35 kg/m^2^, 428 European American trios (Price *et al.*, unpublished data).

Our results showed that rs1526167 was associated with both T2DM and MVCD, although the risk allele was different for those two conditions. The biological connections between *TOX* and T2DM are poorly understood, although *TOX* gene polymorphisms are associated with insulin resistance traits in both Han Chinese (present study) and European Americans.

Since we have not genotyped all reported “positive” SNPs for our candidate genes, we failed to replicate many well established associations. However, the main purpose of this study is not to exclude certain genes from the T2DM/MVCD candidates. We have selected most of our SNPs based on minor allele frequencies (MAF) in Han Chinese, although several SNPs that with higher MAFs in Caucasians were genotyped to verify our previous findings.

In our study, we employed a well-phenotyped, ethnically homogenous population of diabetic subjects, although the number of non-MVCD T2DM controls was relatively limited. A U.S. NHANES epidemiological study showed that prevalence of MVCDs reaches its peak 15 years after onset of T2DM, with almost no new MVCDs developing in individuals with >15 years of T2DM history^28^. In the present study, we considered individuals with a history of T2DM >10 years, without MVCDs, to be unlikely to carry MVCD susceptibility genes. In our study, the duration of T2DM history in non-MVCD controls was 17.0 ± 5.2 years. We therefore selected these individuals as non-MVCD controls. More controls need to be recruited for association studies with larger power, and much more genotyping needs to be done in the subjects to better understand these relationships between SNPs and MVCDs.

## Materials and Methods

### Study population and design

We recruited 1,939 T2DM diabetic patients and 918 individuals >57 years old with normal blood glucose levels as nondiabetic controls. We collected older subjects for controls since the late onset of T2DM. All the subjects were unrelated Han Chinese collected from the Metabolic Disease Hospital of Tianjin Medical University, General Hospital of Tianjin Medical University, Tianjin People’s Hospital, and Eye Hospital of Tianjin Medical University. We examined the patients for DR and DN: 836 patients had DN, 398 had nonproliferative DR (NPDR), 106 had proliferative DR (PDR), and 504 had DR; 224 patients had both DN and DR (612 and 280 subjects only had DN or DR, respectively). Overall, 1,116 patients had MVCD (DN or DR). As non-MVCD controls, we used 266 patients with a history of T2DM for >10 years who never developed DR or DN; the average duration of T2DM history in non-MVCD controls was 17.0 ± 5.2 years (Table 4).

All subjects gave written informed consent prior to this study, and the protocol was approved by the Committee on Studies Involving Human Beings at Tianjin Medical University. The study was carried out in accordance with the approved guidelines.

We collected the patient’s general information and clinical characteristics, including gender, age, height, weight, biochemistry and lipid profiles, and fasting plasma glucose. Patients were examined for DR and DN. All phenotypes were documented in a Filemaker Pro database. Table 4 presents the basic characteristics of the study population.

### Diabetic retinopathy assessment

All patients received a professional fundus examination and fundus photography; the results were checked and graded by two ophthalmologists at the Eye Hospital of Tianjin Medical University. Level of retinopathy was defined according to a new international classification of DR^44^ developed by the 29th International Congress of Ophthalmology in 2002. This classification comprises five levels: non-DR, mild nonproliferative DR, moderate nonproliferative retinopathy, severe nonproliferative retinopathy and proliferative retinopathy (PDR). If the levels of DR were inconsistent for the two eyes, the worse eye was recorded for the patient. We used as cases both all DR patients and the subset of PDR patients for separate analyses (Table 4).

### Diabetic nephropathy measurement

Microalbumin excretion rates were measured for each patient. The diagnostic criteria for DN includes a history of diabetes and microalbumin/creatinine >300 mg/g, or diagnosis by the renal biopsy. All patients with primary glomerular disease and other secondary glomerular diseases were excluded.

### Candidate gene selection and genotyping

As candidate genes for case-control association studies, we selected 1) genes associated with T2DM, obesity, MVCD, or insulin resistance in our^45^ and other previous candidate gene associations and genome wide association studies (GWAS)^21^,^46^,^47^; and 2) genes in biological pathways related to the development of MVCD, glucose and lipid metabolism, or insulin secretion in diabetes. We selected 82 SNPs in 54 candidate genes (Table 3). Minor allele frequencies (MAFs) of Han Chinese, European American, and global populations were taken from dbSNP ( http://www.ncbi.nlm.nih.gov/snp/). For previously reported associations, we selected SNPs with the most significant association rather than genotyping the whole gene. For less studied genes, multiple SNPs were chosen based on the linkage disequilibrium pattern of the gene (D’ > 0.6). In this study, we have not genotyped all tagged SNPs in certain candidate genes due to limited resources. We also performed gene-specific power calculations for *IRS1, TCF7L2, KCNQ1*, and *FTO* genes based on real minor allele frequencies (MAF) in our data set (Supplement Table 1). Linkage disequilibrium among candidate gene SNPs (in D’ and r^2^) was calculated by Haploview^48^, results were shown as Supplement Table 2. Linkage disequilibrium (LD) patterns (in r^2^)of the *TOX* gene region SNPs were shown as Supplement Figure 1 (Chinese, CHB) and Supplement Figure 2 (Caucasian, CEU).

Genomic DNA samples were extracted from peripheral whole blood samples using the high-salt method. Genotyping was performed by primer extension of multiplex products with detection by matrix-assisted laser desorption time-of-flight mass spectrometry.

### Association Studies

The Hardy-Weinberg equilibrium (HWE) test was performed before the association analysis (Table 3). Statistical analyses for phenotypes were performed by SPSS, version 17.0. The allelic frequencies between the case group and the control group were compared by chi tests using PLINK^49^, and odds ratios (ORs) with 95% confidence intervals (CIs) are presented.

Association studies were carried out in two stages: 1) using 1,939 T2DM patients as cases and 918 nondiabetic individuals as controls, to test associations for T2DM; and 2) using 1,116 MVCD patients as cases and 266 patients with a history of T2DM for >10 years, but without MVCDs, as controls, to test associations for MVCDs. Association studies were performed separately for DN, DR, PDR, and MVCD.

## Conclusions

In summary, our case-control studies suggest that *TOX* and *CDKN2A/B* gene SNPs are associated with T2DM, DN, DR, and MVCD in Han Chinese. A large prospective study is needed to confirm these associations in Han Chinese. A better understanding of genetic factors predisposing individuals to diabetic complications would help identify diabetic patients at risk and also to reveal the pathogenesis of MVCD.

## Additional Information

**How to cite this article**: Wei, F. *et al.* *TOX* and *CDKN2A/B* Gene Polymorphisms Are Associated with Type 2 Diabetes in Han Chinese. *Sci. Rep.* **5**, 11900; doi: 10.1038/srep11900 (2015).

## Supplementary Material

Supplementary Information

## Figures and Tables

**Table 1:** Association analyses for T2DM (1939 cases, 918 non-T2DM controls, nominal *P *< 5 × 10^−3^).

**SNP**	**Chr**	**Position**	**Gene**	**Risk allele**	**Risk allele frequencies**	***P***	**OR (95% CI)**
rs10811661	9	22134094	*CDKN2A/B*	T	0.56	4.09 × 10^−7^#	1.364 (1.209 ~ 1.538)
rs1526167	8	59702355	*TOX*	A	0.46	**2.85** × **10**^−9^*	1.440 (1.276 ~ 1.624)
rs4402960	3	185511687	*IGF2BP2*	T	0.25	7.56 × 10^−4^	1.352 (1.134 ~ 1.612)
rs6856526	4	61057462	*LPHN3*	C	0.93	1.33 × 10^−3^	1.522 (1.176 ~ 1.970)
rs13266634	8	118184783	*SLC30A8*	C	0.64	4.56 × 10^−3^	1.282 (1.080 ~ 1.532)
rs7756992	6	20679709	*CDKAL1*	G	0.54	8.21 × 10^−4^	1.246 (1.095 ~ 1.418)

^*^Significant for genome-wide association (*P *< 5 × 10^−8^).

^#^Significant after multiple tests correction.

**Table 2:** Candidate gene association studies for MVCD (266 non-MVCD T2DM patients as controls).

**SNP**	**rs4402960**	**rs1526167**	**rs12102171**	**rs722208**	**rs10811661**
chromosome	3	8	15	6	9
position	185511687	59702355	67425033	152322885	22134094
Gene	*IGF2BP2*	*TOX*	*SMAD3*	*ESR1*	*CDKN2A/B*
Risk allele	G	G	T	A	T
DN (N = 836)					
Risk allele frequencies	0.72	0.48			
*P*	0.0092	0.0011			
OR (95% CI)	1.499 (1.104 ~ 2.034)	1.470 (1.166 ~ 1.854)			
DR (N = 504)					
Risk allele frequencies		0.46	0.31	0.46	0.58
*P*		0.0082	0.027	0.041	0.037
OR (95% CI)		1.412 (1.093 ~ 1.825)	1.471 (1.043 ~ 2.072)	1.305 (1.011 ~ 1.686)	1.314 (1.016 ~ 1.698)
PDR (N = 106)					
Risk allele frequencies				0.44	0.57
*P*				0.033	0.026
OR (95% CI)				1.632 (1.038 ~ 2.566)	1.708 (1.063 ~ 2.743)
MVCD (N = 1116)					
Risk allele frequencies	0.72	0.49	0.31		0.59
*P*	0.0094	4.33 × 10^−4^	0.045		0.025
OR (95% CI)	1.481 (1.100 ~ 1.995)	1.498 (1.195 ~ 1.878)	1.376 (1.007 ~ 1.879)		1.292 (1.032 ~ 1.617)

Only results with nominal *P *< 0.05 are shown.

**Table 3:** Candidate genes and SNPs genotyped in this study.

**SNP**	**Gene**	**Chr**	**HWE (*****P***)	MAF*		
				**CHB**	**CEU**	**Global**
rs7546903	*CAMTA1*	1	0.603	0.463	0.226	0.368
rs1801133	*MTHFR*	1	0.236	0.439	0.310	0.325
rs6427665	*NOS1AP*	1	0.942	0.455	0.233	0.378
rs2661812	*NOS1AP*	1	0.596	0.475	0.500	0.445
rs16867321	*UBE2E3*	2	1.000	0.415	0.200	0.271
rs62183937	*ABI2*	2	1.000	0.475	0.125	0.258
rs11675251	*ABI2*	2	0.303	0.171	0.482	0.381
rs3731652	*ABI2*	2	0.666	0.433	0.158	0.375
rs1376877	*ABI2*	2	0.177	0.171	0.455	0.383
rs11677793	*SPAG16*	2	0.992	0.200	0.456	0.284
rs7578326	*IRS1*	2	0.512	0.125	0.350	0.304
rs1678607	*VHL*	3	0.768	0.111	0.125	0.208
rs13081389	*PPARγ*	3	0.438	0.022	0.042	0.034
rs35747495	*PCAF*	3	0.979	0.325	0.300	0.260
rs2929402	*PCAF*	3	0.227	0.463	0.372	0.419
rs1986917	*PCAF*	3	0.987	0.433	0.442	0.389
rs4402960	*IGF2BP2*	3	0.188	0.256	0.280	0.343
rs13129697	*SLC2A9*	4	0.920	0.439	0.292	0.423
rs1014290	*SLC2A9*	4	0.892	0.363	0.257	0.308
rs6856526	*LPHN3*	4	0.484	0.073	0.009	0.129
rs2231142	*ABCG2*	4	0.989	0.293	0.111	0.139
rs10946398	*CDKAL1*	6	0.851	0.439	0.336	0.408
rs7756992	*CDKAL1*	6	0.312	0.488	0.279	0.405
rs1165196	*SLC17A1*	6	0.205	0.232	0.451	0.260
rs881858	*VEGFA*	6	0.045	0.189	0.292	0.346
rs9395706	*PKHD1*	6	0.989	0.476	0.128	0.296
rs722208	*ESR1*	6	0.583	0.500	0.246	0.412
rs4880	*SOD2*	6	0.713	0.111	0.300	0.188
rs1581498	*SNORD93*	7	0.362	0.400	0.467	0.378
rs1799884	*GCK*	7	0.084	0.171	0.195	0.188
rs705382	*PON1*	7	0.755	0.415	0.336	0.472
rs1007311	*NOS3*	7	0.587	0.308	0.500	0.437
rs768403	*GBX1*	7	0.877	0.463	0.398	0.476
rs7805834	*NUB1*	7	0.694	0.073	0.102	0.140
rs446886	*NUB1*	7	0.151	0.073	0.310	0.298
rs386956	*NUB1*	7	0.972	0.488	0.319	0.478
rs1526167	*TOX*	8	0.445	0.478	0.467	0.478
rs2726557	*TOX*	8	0.254	0.427	0.327	0.459
rs11777927	*TOX*	8	0.757	0.356	0.267	0.356
rs17304270	*TOX*	8	0.130	0.061	0.288	0.390
rs13266634	*SLC30A8*	8	0.930	0.476	0.239	0.282
rs10811661	*CDKN2A/B*	9	0.764	0.415	0.199	0.206
rs3758391	*SIRT1*	10	0.965	0.195	0.270	0.473
rs7923837	*HHEX*	10	1.000	0.244	0.367	0.427
rs7903146	*TCF7L2*	10	0.236	0.024	0.279	0.218
rs2237892	*KCNQ1*	11	0.287	0.317	0.075	0.170
rs2166706	*MTNR1B*	11	0.658	0.317	0.389	0.472
rs189037	*ATM*	11	0.997	0.389	0.485	0.485
rs7312112	*IGF1*	12	0.304	0.500	0.385	0.462
rs2241220	*ACACB*	12	0.996	0.341	0.142	0.109
rs11067076	*TBX5*	12	0.586	0.037	0.257	0.193
rs11067083	*TBX5*	12	0.545	0.064	0.242	0.199
rs371276	*SLITRK5*	13	0.615	0.463	0.025	0.244
rs409762	*SLITRK5*	13	0.832	0.488	0.013	0.244
rs2301113	*HIF1A*	14	0.925	0.317	0.173	0.428
rs11624704	*NRXN3*	14	0.492	0.049	0.137	0.115
rs1498506	*SMAD3*	15	0.840	0.433	0.475	0.455
rs12102171	*SMAD3*	15	0.353	0.341	0.195	0.276
rs17818920	*FTO*	16	1.000	0.183	0.250	0.258
rs1876942	*FTO*	16	0.309	0.341	0.425	0.479
rs708254	*FTO*	16	0.438	0.350	0.420	0.390
rs2239359	*FANCA*	16	0.970	0.207	0.416	0.393
rs7190823	*FANCA*	16	0.488	0.024	0.416	0.343
rs4353	*ACE*	17	0.953	0.317	0.495	0.498
rs17782313	*MC4R*	18	0.489	0.232	0.265	0.221
rs8109627	*CCDC97*	19	0.928	0.390	0.235	0.341
rs4814615	*PCSK2*	20	1.000	0.488	0.128	0.293
rs3746876	*KCNJ15*	21	0.906	0.110	0.004	0.046
rs2106294	*LIMK2*	22	0.824	0.073	0.305	0.178
rs5749286	*SFI1*	22	0.875	0.378	0.283	0.271
rs5753669	*SFI1*	22	0.497	0.378	0.283	0.271
rs2295251	*SFI1*	22	0.652	0.451	0.270	0.404
rs735853	*MYH9*	22	0.991	0.110	0.477	0.273
rs875726	*MYH9*	22	0.456	0.305	0.296	0.449
rs2009930	*MYH9*	22	0.377	0.275	0.283	0.450
rs2239782	*MYH9*	22	0.067	0.317	0.239	0.349
rs3752462	*MYH9*	22	0.320	0.305	0.332	0.453
rs2269532	*MYH9*	22	0.881	0.267	0.358	0.390
rs2071731	*MYH9*	22	0.999	0.280	0.367	0.423
rs739097	*MYH9*	22	0.876	0.268	0.456	0.495
rs2285094	*PDGFB*	22	0.549	0.183	1.000	0.328
rs738409	*PNPLA3*	22	0.804	0.344	0.233	0.284

^*^MAF: minor allele frequencies, taken from dbSNP. CHB, Han Chinese; CEU, European American.

**Table 4:** Basic characteristics of T2DM and MVCD cases and controls.

	**T2DM**	**Non-diabetic controls**	**DN**	**DR**	**PDR**	**MVCD**	**Non-MVCD T2DM controls**
N	1,939	918	836	504	106	1,116	266
Sex (male/female)	1,040/899	740/178	482/354	257/247	44/62	605/511	124/142
Age (yr, mean ± SD)	58.5 ± 12.0	72.4 ± 9.1	58.8 ± 11.1	57.8 ± 11.1	54.6 ± 11.1	58.7 ± 11.1	63.4 ± 9.3
T2DM duration (yr, mean ± SD)	10.3 ± 7.7	N/A	11.3 ± 7.6	12.6 ± 7.6	13.1 ± 8.2	11.6 ± 7.7	17.0 ± 5.2
