# Supplementary material for: TOX and CDKN2A/B Gene Polymorphisms Are Associated with Type 2 Diabetes in Han Chinese
Source: Sci Rep. 2015 Jul 3;5:11900. doi: 10.1038/srep11900 (PMC4650661; doi:10.1038/srep11900)
Supplement: Supplementary Information [file srep11900-s1.pdf]

TOX and *CDKN2A/B* Gene Polymorphisms Are Associated with Type 2 Diabetes in Han Chinese

Running title: *TOX* and *CDKN2A/B* genes are associated with type 2 diabetes

Fengjiang Wei[1*], Chunyou Cai[1*], Shuzhi Feng[2*], Jia Lv[1], Shen Li[1], Baocheng Chang[3], Hong Zhang[4], Wentao Shi[1], Hongling Han[2],Chao Ling[1], Ping Yu[1], Yongjun Chen[4], Ning Sun[2], Jianli Tian[2], Hongxiao Jiao[1], Fuhua Yang[1], Mingshan Li[1], Yuhua Wang[1], Lei Zou[1], Long Su[4], Jingbo Li[5], Ran Li[2], Huina Qiu[5], Jingmin Shi[1], Shiying Liu[2], Mingqin Chang[1], Jingna Lin[5†], Liming Chen[3†], Wei-Dong Li[1†]

[1] Research Center of Basic Medical Sciences, Tianjin Medical University, Tianjin, 300070, China.

[2] Tianjin General Hospital, Tianjin Medical University, Tianjin, 300052, China

[3] Metabolic Diseases Hospital, Tianjin Medical University, Tianjin, 300070, China

[4]Eye Hospital, Tianjin Medical University, Tianjin, 300384, China

[5]Tianjin People's Hospital, Department of Endocrinology, Tianjin, 300191, China

*These authors contributed equally to this manuscript

† *Corresponding author and person to whom reprint requests should be address* to:

Wei-Dong Li, M.D., Ph.D.

Email: liweidong98@tijmu.edu.cn

Tel: 86-22-83336586

Fax: 86-22-23542775

Liming Chen, M.D.

Email: xfx22081@vip.163.com

Tel：86-22-23333266

Fax：86-22-23333269

Jingna Lin, M.D.

Email: linjingna6@yahoo.com

Tel: 86-22-2755-7477

Fax: 86-22-2755-7477

Supplement Table 1 Power calculations for T2DM candidate genes in 1939 cases and 918 controls*

SNP	Gene	Chr	MAF	GRR=1.2			GRR=1.4			GRR=1.6			GRR=1.8		
				0.05	0.01	0.001	0.05	0.01	0.001	0.05	0.01	0.001	0.05	0.01	0.001
rs7578326	*IRS1*	2	0.146	0.346	0.155	0.042	0.814	0.609	0.331	0.976	0.914	0.743	0.998	0.989	0.943
rs7903146	*TCF7L2*	10	0.046	0.111	0.032	0.005	0.258	0.103	0.023	0.435	0.217	0.067	0.597	0.355	0.139
rs2237892	*KCNQ1*	11	0.275	0.479	0.252	0.083	0.942	0.831	0.596	0.998	0.991	0.951	1	1	0.998
rs17818920	*FTO*	16	0.130	0.257	0.102	0.023	0.662	0.422	0.181	0.907	0.760	0.497	0.982	0.930	0.777
rs1876942	*FTO*	16	0.441	0.237	0.091	0.020	0.634	0.401	0.167	0.907	0.761	0.498	0.985	0.941	0.803
rs708254	*FTO*	16	0.444	0.234	0.089	0.020	0.636	0.394	0.163	0.903	0.753	0.488	0.984	0.938	0.794

* Power calculations for T2DM candidate genes were carried out by Genetic Power Calculator (GPC). High risk allele frequency was set equal to 0.25, prevalence=0.1, dominant model, Genotype relative risks were set as 1.2, 1.4, 1.6, and 1.8, respectively. D'=0.9, type I error rates were 0.05, 0.01, and 0.001, respectively.

Supplement Table 2 Linkage disequilibrium among candidate gene SNPs

SNP	chromosome	bp	gene	SNP1	SNP2	D'	r2
rs62183937	2	204193688	ABI2	rs62183937	rs11675251	1	0.444
rs11675251	2	204249399	ABI2	rs62183937	rs3731652	1	0.742
rs3731652	2	204261723	ABI2	rs62183937	rs1376877	0.995	0.431
rs1376877	2	204272090	ABI2	rs11675251	rs3731652	1	0.597
				rs11675251	rs1376877	0.997	0.974
				rs3731652	rs1376877	0.995	0.581
rs4353	17	61570422	ACE	rs4353	rs11868324	0.645	0.126
rs11868324	17	61577309	ACE				
rs10946398	6	20661034	CDKAL1	rs10946398	rs7756992	0.837	0.534
rs7756992	6	20679709	CDKAL1				
rs2239359	16	89849480	FANCA	rs2239359	rs7190823	0.36	0.001
rs7190823	16	89866043	FANCA				
rs1876942	16	54118435	FTO	rs1876942	rs708254	0.985	0.957
rs708254	16	54123389	FTO				
rs35747495	3	20077262	KAT2B	rs35747495	rs1986917	0.024	0
rs1986917	3	20118522	KAT2B				
rs735853	22	36679215	MYH9	rs735853	rs875726	0.836	0.279
rs875726	22	36691969	MYH9	rs735853	rs2009930	0.836	0.278
rs2009930	22	36699306	MYH9	rs735853	rs2239782	0.764	0.225
rs2239782	22	36705104	MYH9	rs735853	rs3752462	0.779	0.25
rs3752462	22	36710183	MYH9	rs735853	rs2269532	0.191	0.02
rs2269532	22	36718039	MYH9	rs735853	rs2071731	0.191	0.02
rs2071731	22	36718858	MYH9	rs735853	rs739097	0.156	0.015
rs739097	22	36746079	MYH9	rs875726	rs2009930	0.997	0.99
				rs875726	rs2239782	0.739	0.529
				rs875726	rs3752462	0.978	0.928
				rs875726	rs2269532	0.512	0.192
				rs875726	rs2071731	0.513	0.193
				rs875726	rs739097	0.544	0.19
				rs2009930	rs2239782	0.736	0.527
				rs2009930	rs3752462	0.982	0.931
				rs2009930	rs2269532	0.517	0.195
				rs2009930	rs2071731	0.518	0.196
				rs2009930	rs739097	0.549	0.193

				rs2239782	rs3752462	0.763	0.546
				rs2239782	rs2269532	0.515	0.188
				rs2239782	rs2071731	0.515	0.189
				rs2239782	rs739097	0.535	0.178
				rs3752462	rs2269532	0.531	0.213
				rs3752462	rs2071731	0.532	0.214
				rs3752462	rs739097	0.57	0.215
				rs2269532	rs2071731	1	1
				rs2269532	rs739097	0.986	0.85
				rs2071731	rs739097	0.986	0.85
rs6427665	1	162214762	NOS1AP	rs6427665	rs2661812	0.308	0.081
rs2661812	1	162281295	NOS1AP				
rs7805834	7	151043272	NUB1	rs7805834	rs446886	1	0.898
rs446886	7	151053043	NUB1	rs7805834	rs386956	0.98	0.116
rs386956	7	151064064	NUB1	rs446886	rs386956	0.982	0.129
rs5749286	22	31900359	SFI1	rs5749286	rs5753669	0.997	0.995
rs5753669	22	31905819	SFI1	rs5749286	rs2295251	0.661	0.203
rs2295251	22	32013265	SFI1	rs5753669	rs2295251	0.658	0.197
rs13129697	4	9926967	SLC2A9	rs13129697	rs7660895	0.733	0.519
rs7660895	4	9985445	SLC2A9	rs13129697	rs1014290	0.93	0.645
rs1014290	4	10001861	SLC2A9	rs7660895	rs1014290	0.94	0.661
rs371276	13	89830501	SLITRK5	rs371276	rs409762	1	0.992
rs409762	13	89840239	SLITRK5				
rs1498506	15	67367634	SMAD3	rs1498506	rs12102171	0.338	0.04
rs12102171	15	67425033	SMAD3				
rs11067076	12	114799863	TBX5	rs11067076	rs11067083	1	0.826
rs11067083	12	114811042	TBX5				
rs1526167	8	59702355	TOX	rs1526167	rs2726557	0.037	0.001
rs2726557	8	59792800	TOX	rs1526167	rs11777927	0.011	0
rs11777927	8	59881039	TOX	rs1526167	rs17304270	0.013	0
rs17304270	8	59979034	TOX	rs2726557	rs11777927	0.244	0.05
				rs2726557	rs17304270	0.498	0.012
				rs11777927	rs17304270	0.38	0.006

D' and r2 were calculated by Haploview

Supplement Figure 1 Linkage disequilibrium (LD) patterns ($\mathbf{r^2}$)of the *TOX* gene region SNPs, Chinese (CHB)

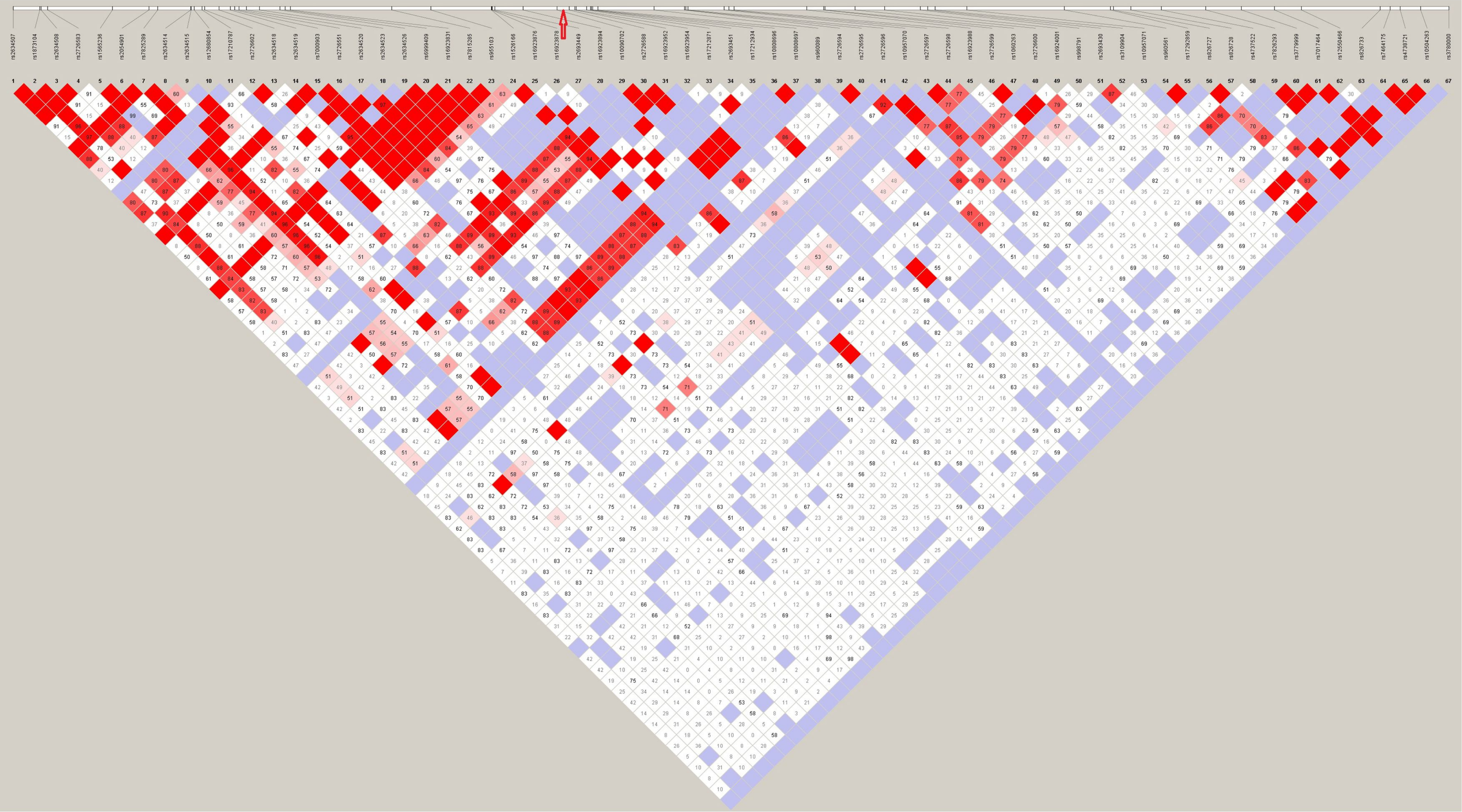

r^2

Wei, et al. *TOX* and *CDKN2A/B* Gene Polymorphisms Are Associated with Type 2 Diabetes in Han Chinese

Supplement Figure 2 Linkage disequilibrium (LD) patterns ($\mathbf{r^2}$) of the *TOX* gene region SNPs (Caucasians, CEU)

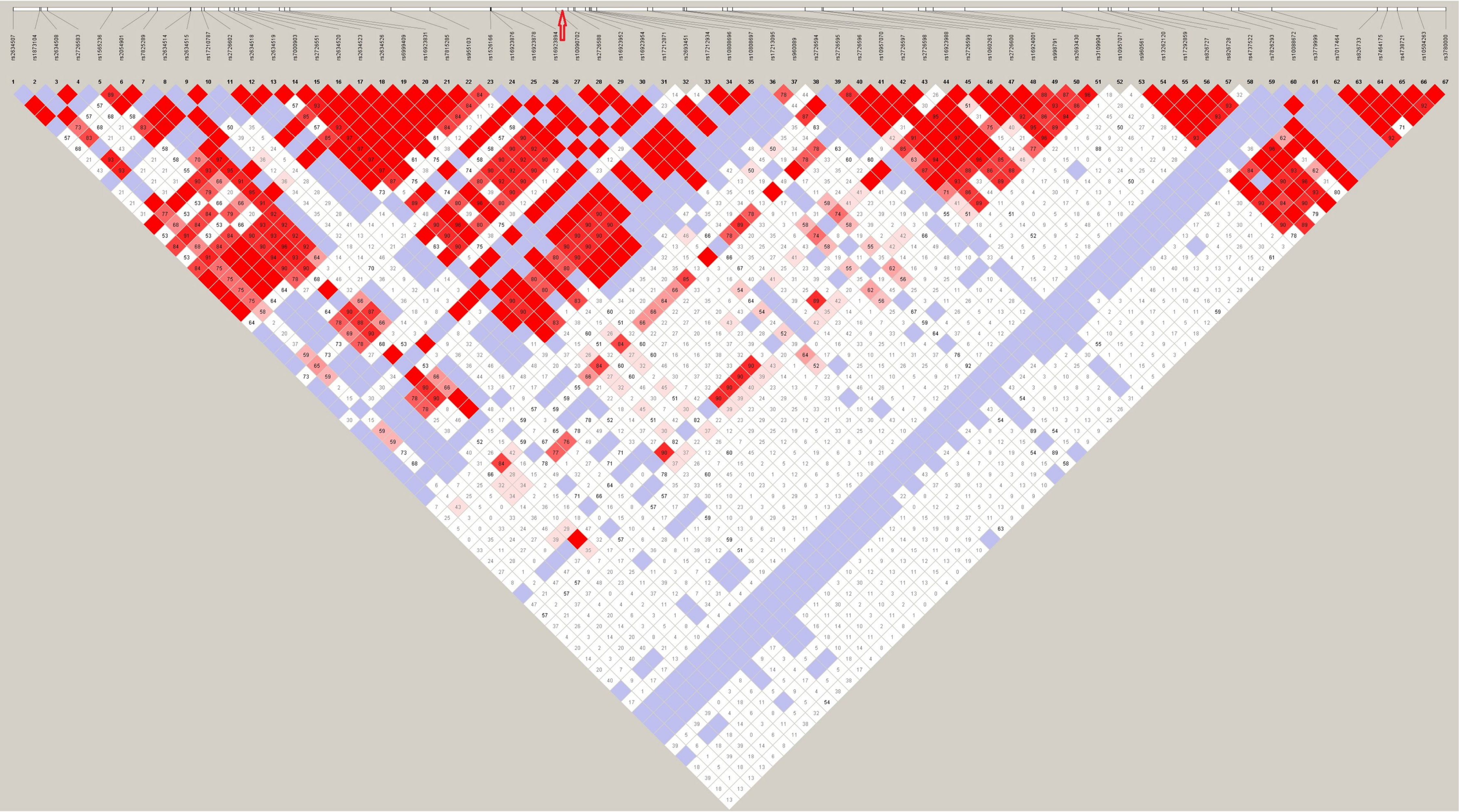

r^2
